# Differences in the incidence of postoperative pneumonia after percutaneous endoscopic gastrostomy between liquid and semi-solid nutrient administration

**DOI:** 10.1038/s41430-018-0380-y

**Published:** 2019-01-04

**Authors:** Hirohito Muramatsu, Tetsuro Okamoto, Tomoko Kubo, Midori Matsuki, Sonomi Iwata, Akemi Fujiwara, Naoya Miyajima, Hidetoshi Inomata, Tomokazu Hoshi, Yoshiro Goto

**Affiliations:** 1 Department of Gastroenterology, Rumoi Municipal Hospital, Rumoi, Japan; 2 Department of Gastroenterology, Sapporo Kiyota Hospital, Sapporo, Japan; 3 Department of Nursing, Sapporo Kiyota Hospital, Sapporo, Japan; 4 Department of Nutrition, Sapporo Kiyota Hospital, Sapporo, Japan; 5 Department of Surgery, Rumoi Municipal Hospital, Rumoi, Japan; 6 Department of Internal Medicine, Sapporo Kiyota Hospital, Sapporo, Japan

**Keywords:** Nutrition, Nutritional supplements

## Abstract

**Background/Objectives:**

This historical control study examined the differences in the incidence of postoperative pneumonia between patients administered liquid and semi-solid nutrients after percutaneous endoscopic gastrostomy (PEG).

**Subjects/Methods:**

The medical records of adult patients who underwent PEG between March 1999 and March 2014 were investigated. The patients were administered either liquid or semi-solid nutrient and examined for gastroesophageal reflux via radiography after PEG. The study period was divided into periods I (liquid nutrient to all patients), II (semi-solid nutrient to patients with reflux and liquid nutrient to those without), and III (semi-solid nutrient to all patients). The patient characteristics and incidence of postoperative pneumonia were stratified by the periods. To assess the relationship between postoperative pneumonia and the periods, a logistic regression analysis was performed.

**Results:**

Of 370 patients enrolled, 149 were in period I, 64 in period II, and 157 in period III. Postoperative pneumonia was more frequently observed in period I (20.8%) than in periods II (7.8%) and III (10.2%). The odds ratios were higher in period I (period I vs. II: 3.10 [95% confidence intervals: 1.15–8.38]; period I vs. III: 2.32 [1.21–4.44]). The incidence of gastroesophageal reflux did not greatly differ between periods II (25.0%) and III (35.0%).

**Conclusions:**

The incidence of postoperative pneumonia after PEG was lower in the patients administered semi-solid nutrient than in those administered liquid nutrient, suggesting that semi-solid nutrient administration to patients with PEG tubes is preferable to prevent postoperative pneumonia. Furthermore, it may be favored especially in those with gastroesophageal reflux.

## Introduction

Percutaneous endoscopic gastrostomy (PEG) is considered a useful method of feeding patients with impaired oral intake resulting from disorders, including cerebrovascular accident. The guidelines on enteral nutrition recommend the use of PEG for physiological management of the nutrition for such patients [1–3]. Meanwhile, PEG is related to the risk of common complications, such as peristomal infection, and major severe complications, such as aspiration pneumonia, bleeding, buried bumper syndrome, and bowel perforation [4–6]. Particularly, pneumonia mainly caused by aspiration after PEG can be fatal [7–9]. Up to 50 % of postoperative early mortality within 30 days after PEG was attributable to aspiration pneumonia [8, 9].

Aspiration after PEG is mainly induced by throat secretions or refluxed stomach contents, such as nutrients [10, 11]. Patients with gastroesophageal reflux are reportedly more likely to have postoperative pneumonia after PEG than those without [12]. For prevention of aspiration caused by gastroesophageal reflux, it is suggested to optimize the amount and speed of nutrient administration, adjust patient positioning, and use prokinetic agents [13, 14]. In Japan, in the expectation of preventing aspiration caused by reflux of nutrients in patients with PEG tubes, the use of semi-solid nutrient instead of liquid nutrient has been advocated [15, 16]. Semi-solid nutrient administration is increasingly reported to be associated with a lower incidence of aspiration pneumonia after PEG than liquid nutrient administration [17, 18]. However, evidence on the relationships between semi-solid nutrient administration and aspiration pneumonia is still lacking.

Therefore, in this study, we examined the differences in the incidence of postoperative pneumonia between patients administered liquid nutrient and those administered semi-solid nutrient after PEG.

## Subjects and methods

### Study design

This was an observational retrospective historical control study using the medical records of patients who underwent PEG in the Department of Gastroenterology of Sapporo Kiyota Hospital (Sapporo, Hokkaido, Japan) between March 1999 and March 2014.

The study period was divided into three periods according to the nutrient forms administered. In period I, liquid nutrient was administered to all patients after PEG between March 1999 and June 2005. In period II, liquid nutrient was administered to patients who had no gastroesophageal reflux after PEG, while semi-solid nutrient was administered to patients who had gastroesophageal reflux after PEG between November 2005 and May 2007. In period III, semi-solid nutrient was administered to all patients after PEG between April 2010 and March 2014.

### Study population

This study included patients who underwent PEG at the hospital between March 1999 and March 2014 and were aged ≥ 20 years at the time of the implementation of PEG. The following patients were excluded from this study: patients who underwent PEG between July and October 2005 (the nutrient management methods were changed); who underwent PEG between June 2007 and March 2010 (they participated in another clinical study); who did not undergo gastrointestinal contrast radiography within 2 days after PEG; who underwent PEG for decompression; whom physicians considered inappropriate for the study; who had gastroesophageal reflux but were administered liquid nutrient during period II; and who had no gastroesophageal reflux but were administered semi-solid nutrient during period II.

### Dose and semi-solid feeding

The nutrient and water were first administered to the patients through gastrostomy on the next day after PEG. The target amount of feeding calories was from 1000 to 1200 kcal (4186 to 5023 kJ) per day, administered in three divided doses. The amount of water including the water contained in the nutrient was set from 1400 to 1600 ml. The amounts of both nutrient and water were adjusted on the basis of the patients’ body size.

In period I, we prescribed an elemental and polymeric liquid diet. In period II, a polymeric liquid diet was prescribed for those who were administered liquid nutrients. The semi-solid nutrient used in period II was either one prepared by mixing a polymeric liquid diet with a gelling agent (Easygel^®^; Otsuka Pharmaceutical Factory, Inc., Tokushima, Japan, viscosity of the mixture: 20,000 mPa s, at 20 °C, 12 rpm) or Hine^®^ Jelly Aqua (Otsuka Pharmaceutical Factory, Inc., viscosity: 6000 mPa s, at 20 °C, 12 rpm); for the water supply, semi-solid water agar was used. In period III, the semi-solid nutrient, Hine^®^ Jelly Aqua or Recovery New Treat^®^ (Nutri Co., Ltd., Mie, Japan, viscosity: 5000 mPa s, at 25 °C, 12 rpm), was used; for the water supply, New Treat^®^ Water (Nutri Co., Ltd.) was used. Each administration of the semi-solid nutrients was assisted by a nurse for ~10 min.

### Data collection

The following data of each patient were extracted from the medical records: age, sex, underlying disease, prognostic nutritional index proposed by Onodera et al. [19], year and month of PEG, PEG method (pull or introducer method), form of nutrient (liquid or semi-solid), and presence or absence of gastroesophageal reflux.

Gastroesophageal reflux was confirmed via gastrointestinal contrast radiography within 2 days after PEG. For gastrointestinal contrast radiography, 100 ml of a radiographic contrast agent (Gastrografin^®^: Bayer Yakuhin, Ltd., Osaka, Japan) diluted with 100 ml of water was administered through gastrostomy to the patients in the supine position. To confirm the presence of gastroesophageal reflux, the reflux of the contrast agent to the esophagus was observed for ≥1 min, and even a small amount of contrast agent reflux was considered to indicate the presence of reflux.

### Endpoints

Our primary endpoint was postoperative pneumonia, which was defined as pneumonia developing within 14 days after PEG accompanied with the following findings: body temperature of ≥37.5 °C; respiratory symptoms; abnormal blood test results, including white blood cell count and C-reactive protein level; and infiltrative shadow observed on chest radiography or chest computed tomography.

Our secondary endpoints were postoperative 30-day mortality, peristomal leakage, and peristomal infection. Postoperative 30-day mortality was defined as any mortality occurring within 30 days after PEG. Peristomal leakage was defined as adherence of nutrients on cotton or tissue paper found during nursing care, while peristomal infection was defined as purulent discharge around the PEG tube found during nursing care. Both of them were monitored for 14 days after PEG.

### Statistical analysis

The demographic and clinical characteristics of all patients were descriptively summarized. The frequency of endpoints was summarized for each period. Continuous variables were expressed as means and standard deviations and categorical variables as numbers and percentages.

Logistic regression analyses were performed to assess the relationship between the endpoints and each period. Odds ratios (ORs) of the incidence of the endpoints among the periods and their 95% confidence intervals (CIs) were calculated.

An exploratory analysis of postoperative pneumonia was also performed. The patient characteristics (i.e., age, sex, underlying disease, prognostic nutritional index, and PEG method) stratified by the presence of postoperative pneumonia were summarized to identify the possible risk factors for postoperative pneumonia. Chi-square test or *t*-test was performed to compare the groups with and without postoperative pneumonia. The variables with *P* < 0.05 were selected as the possible risk factors for postoperative pneumonia and included in the logistic regression model. The ORs of the incidence of postoperative pneumonia among the periods and their 95% CIs, adjusted for the possible risk factors, were calculated.

The test was two-tailed, with the statistical significance level set at *P* < 0.05. No imputation for missing data was performed. All the analyses were performed using SAS version 9.4 (SAS Institute, Inc., Cary, NC, USA). The data were monitored and statistically analyzed independently by Clinical Study Support, Inc. (Nagoya, Aichi, Japan).

### Ethical statement

This study conducted in accordance with the Declaration of Helsinki was approved by the ethical committee of Sapporo Kiyota Hospital; the study protocol prepared before the study was registered in the University Hospital Medical Information Network Center (UMIN000031294). Obtaining of consent from the patients was not required because no invasive procedure, intervention, or human samples were used in this study. This was compliant with the Japanese Ethical Guidelines for Medical and Health Research Involving Human Subjects [20], which do not require informed consent from patients enrolled in studies exclusively utilizing anonymized data. However, we provided opportunities to the subjects to opt out of the study by announcing the study information on the bulletin boards in the hospital and the hospital website.

## Results

### Patient characteristics

**Fig. 1:**
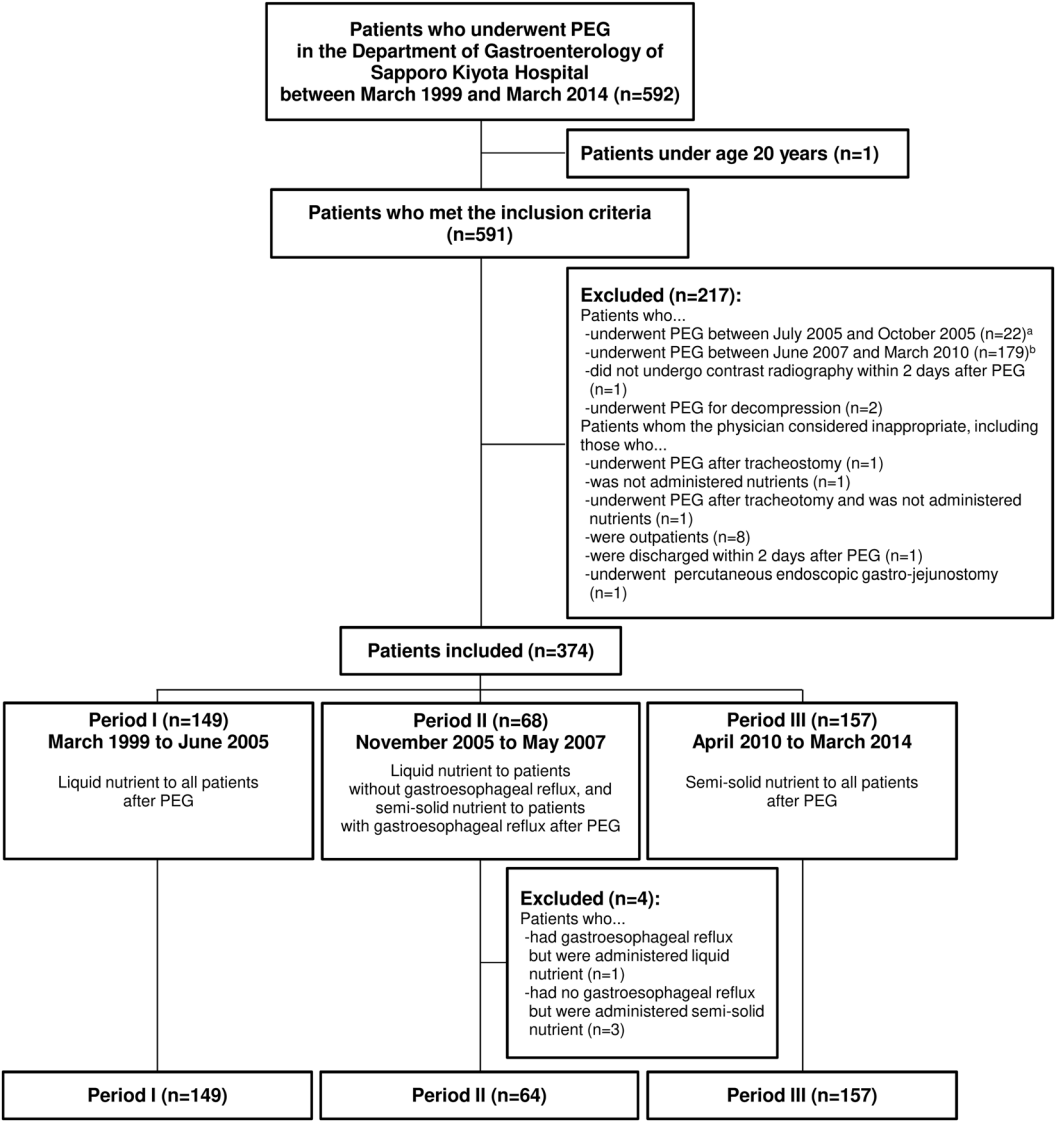
Patient flow diagram. ^a^The nutrient management methods were changed during this period. ^b^Patients participated in another clinical study during this period. PEG percutaneous endoscopic gastrostomy

A total of 370 out of 592 patients who underwent PEG at the hospital during the study period were included in this study (Fig. 1). Of them, 149 were in period I, 64 in period II, and 157 in period III.

Table 1 summarizes the demographic and clinical characteristics of the patients. Their mean age was approximately 77 years in all three periods. The female patients accounted for over half of the total patients in period I (57.7%); their proportion was lower in periods II (45.3%) and III (44.6%). More patients had dementia in period I (13.4%) than in periods II (1.6%) and III (3.2%). Gastroesophageal reflux was most frequently observed in period I (43.0%), followed by periods III (35.0%) and II (25.0%). The pull method was most frequently applied in period I (99.3%) and the introducer method in period III (88.5%).

**Table 1:** Patient characteristics

	Period I (*n* = 149) *n* (%)	Period II (*n* = 64) *n* (%)	Period III (*n* = 157) *n* (%)
Age (years), mean ± SD	77.4 ± 10.6	77.5 ± 11.9	77.5 ± 10.9
*Sex*			
Male	63 (42.3)	35 (54.7)	87 (55.4)
Female	86 (57.7)	29 (45.3)	70 (44.6)
*Underlying disease*			
Cerebrovascular disease	112 (75.2)	44 (68.8)	118 (75.2)
Dementia	20 (13.4)	1 (1.6)	5 (3.2)
Parkinson’s disease	7 (4.7)	2 (3.1)	12 (7.6)
Other	10 (6.7)	17 (26.6)	22 (14.0)
PNI, mean ± SD	42.9 ± 6.7	41.0 ± 5.0	41.3 ± 5.2
*PEG method*			
Introducer method	1 (0.7)	29 (45.3)	139 (88.5)
Pull method	148 (99.3)	35 (54.7)	18 (11.5)
*Form of nutrient*			
Liquid	149 (100.0)	48 (75.0)	0 (0.0)
Semi-solid	0 (0.0)	16 (25.0)	157 (100.0)
*Gastroesophageal reflux* ^a^			
Without	85 (57.0)	48 (75.0)	101 (64.3)
With	64 (43.0)	16 (25.0)	55 (35.0)

Percentages may not add up to 100% because of rounding

*SD* Standard deviation, *PNI* prognostic nutritional index, *PEG* percutaneous endoscopic gastrostomy

^a^One patient was excluded from period III because the results of gastrointestinal contrast radiography were unknown

### Incidence of postoperative pneumonia

Postoperative pneumonia was more frequently observed in period I (20.8%) than in periods II (7.8%) and III (10.2%) (Table 2). The ORs of postoperative pneumonia for each period estimated using the logistic regression model were 3.10 (95% CI: 1.15–8.38) for period I vs. II, 2.32 (1.21–4.44) for period I vs. III, and 0.75 (0.26–2.13) for period II vs. III.

**Table 2:** Incidence of postoperative pneumonia in each period and the odds ratios compared by periods

	Period I (*n* = 149) *n* (%)	Period II (*n* = 64) *n* (%)	Period III (*n* = 157) *n* (%)
*Postoperative pneumonia*			
Without	118 (79.2)	59 (92.2)	141 (89.8)
With	31 (20.8)	5 (7.8)	16 (10.2)

	OR	95% CI
*Postoperative pneumonia*		
Period I vs. II	3.10	1.15‒8.38
Period I vs. III	2.32	1.21‒4.44
Period II vs. III	0.75	0.26‒2.13

*OR* Odds ratio, *CI* confidence interval

The exploratory analysis showed that the underlying disease and PEG method may be the potential risk factors for postoperative pneumonia (*P* < 0.05) (Table 3). These two potential risk factors were included in the logistic regression model. The ORs of postoperative pneumonia for each period were 2.72 (0.85–8.72) for period I vs. II, 2.66 (0.74–9.61) for period I vs. III, and 0.98 (0.30–3.23) for period II vs. III.

**Table 3:** Patient characteristics stratified by the presence of postoperative pneumonia and the adjusted odds ratios compared by periods

	Postoperative pneumonia		*P*-value^a^
	Without (*n* = 318) *n* (%)	With (*n* = 52) *n* (%)	
Age, mean ± SD	77.1 ± 11.3	79.9 ± 8.0	0.09
*Sex*			
Male	158 (49.7)	27 (51.9)	0.77
Female	160 (50.3)	25 (48.1)	
*Underlying disease*			
Cerebrovascular disease	237 (74.5)	37 (71.2)	0.006*
Dementia	20 (6.3)	6 (11.5)	
Parkinson’s disease	14 (4.4)	7 (13.5)	
Other	47 (14.8)	2 (3.8)	
PNI, mean ± SD	42.0 ± 5.8	40.9 ± 6.3	0.19
*PEG method*			
Introducer method	152 (47.8)	17 (32.7)	0.04*
Pull method	166 (52.2)	35 (67.3)	

	OR	95% CI
*Postoperative pneumonia*		
Period I vs. II	2.72	0.85‒8.72
Period I vs. III	2.66	0.74‒9.61
Period II vs. III	0.98	0.30‒3.23

*SD* Standard deviation, *PNI* prognostic nutritional index, *PEG* percutaneous endoscopic gastrostomy, *OR* odds ratio, *CI* confidence interval

*P < 0.05

^a^*P*-values were calculated using the *χ*^2^ test or *t*-test, as appropriate.

### Incidence of postoperative 30-day mortality, peristomal leakage, and peristomal infection

As shown in Table 4, four patients (1.1%) died within 30 days after PEG. Peristomal leakage occurred in 6.0% of the patients in period I, 1.6% in period II, and 1.9% in period III. No great difference was observed in the incidence of postoperative 30-day mortality and peristomal leakage among the three periods. The incidence of peristomal infection tended to decrease from period I (28.2%) to period III (12.7%), and there was a significant difference between periods I and III (OR: 2.69, 95% CI: 1.49–4.85).

**Table 4:** Incidence of postoperative 30-day mortality, peristomal leakage, and peristomal infection in each period and the odds ratios compared by periods

	Period I (*n* = 149) *n* (%)	Period II (*n* = 64) *n* (%)	Period III (*n* = 157) *n* (%)
*Postoperative 30-day mortality*			
Without	146 (98.0)	64 (100)	156 (99.4)
With	3 (2.0)	0 (0.0)	1 (0.6)
*Peristomal leakage*			
Without	140 (94.0)	63 (98.4)	154 (98.1)
With	9 (6.0)	1 (1.6)	3 (1.9)
*Peristomal infection*			
Without	107 (71.8)	53 (82.8)	137 (87.3)
With	42 (28.2)	11 (17.2)	20 (12.7)

	OR	95% CI
*Postoperative 30-day mortality*		
Period I vs. II	>9999.99	<0.01–>9999.99
Period I vs. III	3.21	0.33–31.16
Period II vs. III	<0.01	<0.01–>9999.99
*Peristomal leakage*		
Period I vs. II	4.05	0.50–32.63
Period I vs. III	3.30	0.88–12.43
Period II vs. III	0.82	0.08–7.98
*Peristomal infection*		
Period I vs. II	1.89	0.90–3.97
Period I vs. III	2.69	1.49–4.85
Period II vs. III	1.42	0.64–3.17

*OR* Odds ratio, *CI* confidence interval

## Discussion

The differences in the incidence of pneumonia after PEG between liquid and semi-solid nutrient administration were retrospectively examined using the medical records of the patients who underwent PEG at Sapporo Kiyota Hospital between March 1999 and March 2014. After PEG, the incidence of postoperative pneumonia was lower when the patients with gastroesophageal reflux were administered semi-solid nutrient and those without were administered liquid nutrient (period II) and when all patients were administered semi-solid nutrient (period III) than when all patients were administered liquid nutrient (period I).

Our results are consistent with those of previous studies in that the incidence of postoperative pneumonia was lower in patients administered semi-solid nutrient than in those administered liquid nutrient (period III: 10.2% vs. period I: 20.8%, Table 2). In a previous multicenter randomized study, aspiration pneumonia occurred less frequently in patients administered semi-solid nutrient than in those administered liquid nutrient within 2 weeks after PEG (1.3% vs. 14.5%) [17]. Similar results were reported by another previous quasi-randomized study in which a significantly lower incidence of feeding-related aspiration pneumonia was observed in patients administered semi-solid nutrient after PEG than in those administered liquid nutrient (2.2% vs. 22.2%) [18]. These results suggest that semi-solid nutrient administration may be associated with a lower incidence of postoperative pneumonia. Because postoperative pneumonia after PEG is primarily caused by aspiration mainly from gastroesophageal reflux, the prevention of gastroesophageal reflux would be the key to prevent postoperative pneumonia after PEG. In a previous randomized study where patients with PEG tubes were examined for gastroesophageal reflux, the reflux occurred more frequently when only a liquid contrast agent was used than when only a semi-solid agent was employed (27% vs. 11%) [21]. Semi-solid form has higher viscosity and lower fluidity than liquid form; the administration of such reduces the incidence of gastroesophageal reflux and consequently postoperative pneumonia after PEG. Therefore, semi-solid nutrients may be preferable for patients with PEG tubes to prevent postoperative pneumonia.

In our study, the incidence of gastroesophageal reflux confirmed before administration of nutrient did not greatly differ between periods II and III; there was also no great difference in the incidence of postoperative pneumonia between those periods, although liquid nutrient was administered to those without gastroesophageal reflux in period II. Based on these results, it seems that semi-solid nutrient administration may help prevent postoperative pneumonia especially in patients with gastroesophageal reflux. To examine this further, we exploratorily calculated the incidence of postoperative pneumonia in the patients with and without gastroesophageal reflux (Supplementary Table 1). The incidence of postoperative pneumonia in the patients with gastroesophageal reflux was 32.8%, 6.3%, and 9.1% in periods I, II, and III, respectively. It occurred less frequently when semi-solid nutrient was used than when only liquid nutrient was used. Contrarily, the incidence of postoperative pneumonia did not greatly differ in the patients without gastroesophageal reflux among the periods (periods I, II, and III: 11.8%, 8.3%, and 10.9%, respectively). These results support our assumption that semi-solid nutrient administration may be effective especially in patients with gastroesophageal reflux. Shimoyama et al. [22] reported that semi-solid nutrients accelerate antral motility and gastric emptying more than liquid nutrients. Furthermore, semi-solid nutrients, which have a better motility in the stomach in patients with PEG tubes, stay in the proximal stomach for a shorter period than liquid nutrients, possibly lowering the risk for gastroesophageal reflux [16]. Considering these reports and our results, semi-solid nutrient administration may be preferable especially for patients with gastroesophageal reflux after PEG. However, in cases where it is not clear whether a patient has gastroesophageal reflux (e.g., gastrointestinal contrast radiography cannot be performed), semi-solid nutrients may be preferred.

Our results showed no significant difference in the incidence of postoperative 30-day mortality and peristomal leakage among the periods. However, the incidence of peristomal infection decreased from period I to III. Peristomal infection is the most common minor complication after PEG [4–6]. The use of semi-solid nutrient instead of liquid nutrient may reduce the incidence of peristomal infection. However, according to a meta-analysis, the pull method in which the gastric tube is inserted from the mouth, receiving oral bacteria before reaching the PEG site, is associated with a higher risk for infection than the introducer method where the tube is inserted percutaneously [23]. Because the pull method was applied to almost all patients in period I and to over half in period II, it may have affected the occurrence of peristomal infection. In addition, given that no significant difference was observed in the incidence of peristomal infection between the patients administered liquid (10.5%) and semi-solid nutrients (9.3%) in a previous randomized study applying both pull and introducer methods [17], it can be considered that the difference in the incidence of such an infection may probably be attributed to the different PEG methods used.

To our knowledge, this is the first study to examine the difference in the incidence of postoperative pneumonia between patients administered liquid and semi-solid nutrients who underwent PEG in consideration of the presence of gastroesophageal reflux. Our findings will help prevent postoperative pneumonia after PEG in the general population, but more especially in patients with gastroesophageal reflux.

There are some limitations in this study. First, the patient characteristics and healthcare environments may have differed among the periods, which may have affected the incidence of postoperative pneumonia. For example, the patients may have been referred to Sapporo Kiyota Hospital from different types of hospitals depending on the period. Furthermore, the underlying diseases of the patients also differed among the periods. In addition, the healthcare environments should have changed over 10 years of the study period. During this period, the physicians and PEG methods may have varied; particularly, the method shifted to the introducer method from period I to period III. Postoperative nursing care, including oral care, may also have changed. Therefore, these points could be our limitations, and our results should be interpreted with care. However, the possible risk factors for pneumonia (i.e., underlying diseases and PEG methods) were estimated from the patient characteristics and exploratorily analyzed using the logistic regression model to control the bias. The results of the exploratory analysis, showing the same tendency as the primary endpoint, should have supported the results of the primary endpoint. Second, data on patient medical history, including the risk factors for postoperative pneumonia, such as history of pneumonia and esophageal hiatal hernia [13, 24, 25] were not collected. Although assessing gastroesophageal reflux should have covered the lack of such data to some extent because many patients with hiatal hernia tend to have reflux symptoms [21, 26], the relationship between nutrient forms and medical history of pneumonia, which may affect the incidence of postoperative pneumonia, was not assessed. Third, the observation period was short; therefore, the long-term prognosis was not evaluated. Prospective studies that would evaluate the long-term prognosis are needed to investigate the relationships between semi-solid nutrient administration and pneumonia development further.

In conclusion, it is suggested to administer semi-solid nutrient rather than liquid nutrient to patients with PEG tubes to prevent postoperative pneumonia. Based on the results of our exploratory analysis of the incidence of postoperative pneumonia in consideration of the presence of gastroesophageal reflux in addition to the forms of nutrients, the administration of semi-solid nutrient may be favored especially in patients with gastroesophageal reflux after PEG.

## Supplementary information

Supplementary Table 1

Supplementary Table 2

## Data Availability

All data generated or analysed during this study are included in this published article and its supplementary information files.
