# Supplementary material for: Differences in the incidence of postoperative pneumonia after percutaneous endoscopic gastrostomy between liquid and semi-solid nutrient administration
Source: Eur J Clin Nutr. 2019 Jan 4;73(2):250–7. doi: 10.1038/s41430-018-0380-y (PMC6368559; doi:10.1038/s41430-018-0380-y)
Supplement: Supplementary file 1 — Supplementary Table 1 [file 41430_2018_380_MOESM1_ESM.docx]

Supplementary Table 1. Incidence of postoperative pneumonia in the patients with and without gastroesophageal reflux in each period

|  |  | Period I  (n=149) | |  | Period II  (n=64) | |  | Period III^a^  (n=156) | | |
| --- | --- | --- | --- | --- | --- | --- | --- | --- | --- | --- |
|  |  | Postoperative pneumonia | | | | | | | |  |
|  |  | Without | With |  | Without | With |  | Without | With | |
|  |  | n (%) | n (%) |  | n (%) | n (%) |  | n (%) | n (%) | |
| Gastroesophageal reflux |  |  |  |  |  |  |  |  |  | |
| Without |  | 75 (88.2) | 10 (11.8) |  | 44 (91.7) | 4 (8.3) |  | 90 (89.1) | 11 (10.9) | |
| With |  | 43 (67.2) | 21 (32.8) |  | 15 (93.8) | 1 (6.3) |  | 50 (90.9) | 5 (9.1) | |

^a^One patient was excluded because the results of gastrointestinal contrast radiography were unknown.

Percentages may not add up to 100% because of rounding.
